# Localization of mechanical and electrical defects in dry-type transformers using an optimized acoustic imaging approach

**DOI:** 10.1371/journal.pone.0294674

**Published:** 2023-11-17

**Authors:** Zhanxi Zhang, Youyuan Wang, Zhihe Li, Jinzhan Liu

**Affiliations:** 1 State Key Laboratory of Power Transmission Equipment Technology, Chongqing University, Chongqing, People’s Republic of China; 2 Quanzhou Power Supply Company of State Grid Fujian Electric Power Co. Ltd., Quanzhou, People’s Republic of China; Vardhaman College of Engineering, INDIA

## Abstract

This paper presents an acoustic imaging localization system designed to pinpoint common defects in dry-type transformers by analyzing the unique sounds they produce during operation. The system includes an optimized microphone array and an improved multiple signal classification algorithm. Sound signal characteristics of typical defects, such as foreign object intrusion, screw loosening, and partial discharge, are investigated. A 64-element, 8-arm spiral microphone array is designed using a particle swarm optimization algorithm. The multiple signal classification algorithm enhances acoustic imaging quality in field environments by transforming the input from time-domain to preprocessed frequency-domain signals. The power spectra of subarray and main array are combined, forming the optimization algorithm’s output. Experimental results demonstrate the system’s effectiveness and accuracy.

## 1. Introduction

Dry-type transformers are widely utilized in power systems owing to their compact structure, reduced operational losses, safety, and reliability [1, 2]. Although many high-performance intelligent insulation materials have been developed and extensively employed [3, 4], defects will still arise in dry-type transformers during their operation [5, 6]. These defects could escalate into severe malfunctions, jeopardizing the safety of the power system [7]. Hence, early defect detection is paramount.

Dry-type transformers primarily experience faults such as abnormal fan operation, poor contact between dynamic and static tap changer contacts, multiple grounding points in the iron core, interturn short circuits in the windings, and moisture in the insulation [8, 9]. Intriguingly, early-stage defects produce sound signals distinct from those in regular operation [10–12]. Given the simpler structure and sound propagation mode of dry-type transformers compared to oil-immersed variants [13], there is burgeoning interest in harnessing these sound signals for early defect detection.

Defect localization plays a pivotal role in defect diagnosis, especially in the context of dry-type transformers. Currently, the classification and diagnosis of defects in dry transformers can typically be achieved using signals collected by a single sensor [14]. However, defect localization poses significant challenges. To address this issue, recent advancements in acoustic imaging, employing arrays of multiple microphones [15, 16], have shown great potential in machinery fault diagnosis and sound source localization [17–19]. Recognizing the applicability of this technique, acoustic imaging technology has gained traction for locating and diagnosing defects in dry-type transformers [10–12].

For instance, Bao et al. simulated anomalous vibration defects in a dry-type transformer within an acoustic laboratory, and they successfully localized the defects using a deconvolutional beamforming algorithm after collecting acoustic signals from a 112-element microphone array [12]. Building upon this work, Shao et al. extended the approach to include simulations of partial discharge defects, achieving even more accurate results through the utilization of the deconvolutional beamforming algorithm [11]. In another study, Yu et al. conducted simulations of transformer discharge defects in an acoustic laboratory and effectively located the sources of partial discharge using the grid-moving equivalent source method [10].

Despite these advancements, few studies focus on microphone arrays for abnormal noise-type defects in dry-type transformers. Challenges include limited studied defect types, sub-optimal array designs for specific sound characteristics, and the difficulty of existing algorithms in noisy environments.

A series of key technical advancements addressing early noise-type defects in dry-type transformers is presented in this paper. Initially, a dedicated simulation platform for common defects observed in 10kV dry-type transformers was developed. Utilizing this platform, an exhaustive study was conducted on the characteristics of abnormal sound signals. The design of a 64-element, 8-arm spiral microphone array, particularly adept at capturing and analyzing such signals, was derived from this in-depth research. To further enhance the accuracy of sound localization, the particle swarm optimization (PSO) algorithm was employed to refine the configuration of the microphone array. Concurrently, the challenges posed by low signal-to-noise ratios in real-world scenarios were deeply explored, and the existing acoustic imaging algorithms were innovatively improved by integrating time-frequency transformation and sub-array power spectrum superposition techniques. The experimental results demonstrate that various typical defects of dry transformers can be accurately and stably located by our approach, which holds great significance for ensuring the safety of the power system.

## 2. Typical defects simulation

**Fig 1:**
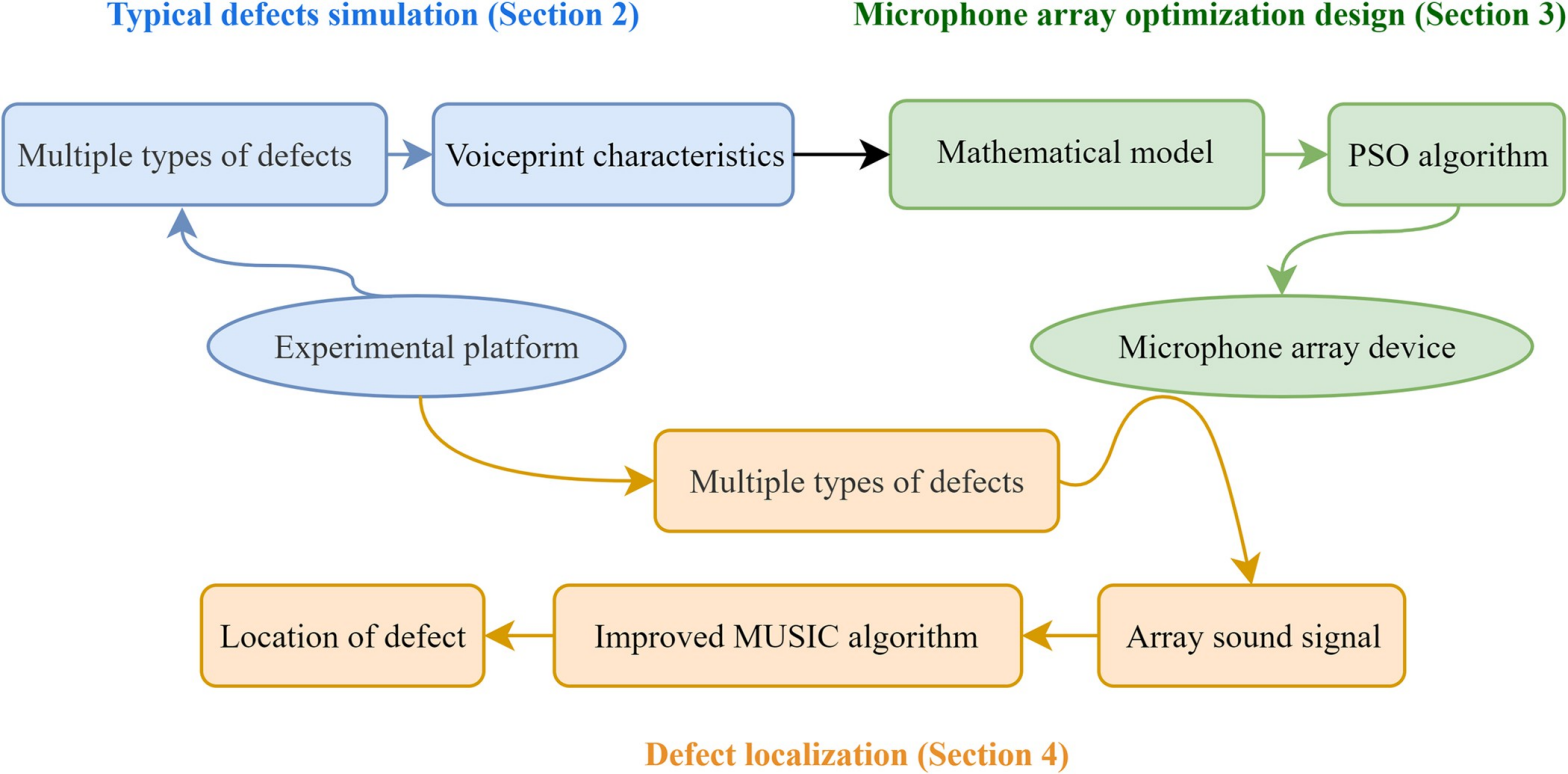
Flowchart of the methodology.

As shown in Fig 1, the methodology of this paper can be divided into three main steps:

1. Utilizing dry-type transformers to construct various defect simulation platforms and performing voiceprint characteristic analysis by collecting acoustic signals from different defects. This corresponds to Section 2 of the paper.
2. Based on the features of the defect acoustic signals (Section 2), guiding the establishment of mathematical models in the microphone array optimization design process. The PSO algorithm is then employed to solve the mathematical model, and the microphone array prototype is subsequently developed based on the solution. This is the focus of Section 3.
3. Using the previously constructed experimental platform (Section 2) and the developed microphone array (Section 3), acoustic array signals from various typical defects are collected. After improving the MUSIC algorithm, higher precision defect localization is achieved. This is detailed in Section 4.

### 2.1 Experimental platform

Based on existing literature, common faults related to sound in dry-type transformers are summarized in Table 1 [8]. For winding short circuits, the most real-time current should be used as the monitoring quantity to ensure equipment safety. For poor contact, as the sound signal produced is discontinuous, temperature should be used as the monitoring quantity; insulation breakdown will directly lead to the cessation of the dry-type transformer’s operation. These faults are not suitable for detection using a microphone array. In contrast, the remaining faults are relatively minor, with good sound continuity, making them suitable for microphone array detection. Therefore, this section simulates three defects: foreign object intrusion into the fan, screw loosening, and partial discharge, to emulate the remaining three types of faults.

**Table 1:** Common dry-type transformer faults related to sound and their handling methods.

Common faults related to sound	Sound characteristics	Severity	Applicability of microphone array
Winding short circuit	Sound similar to boiling	Severe	Not suitable
Insulation breakdown	Strong sound, accompanied by explosive noise	Severe	Not suitable
Poor contact	Discontinuous abnormal noise	Mild	Not suitable
Foreign object intrusion into the fan	Continuous metal friction sound	Mild	Suitable
Loose fixtures or iron cores	Significantly higher than the normal operating sound, and irregular	Mild	Suitable
Partial discharge	Continuous "crackle" discharge sound	Mild	Suitable

**Fig 2:**
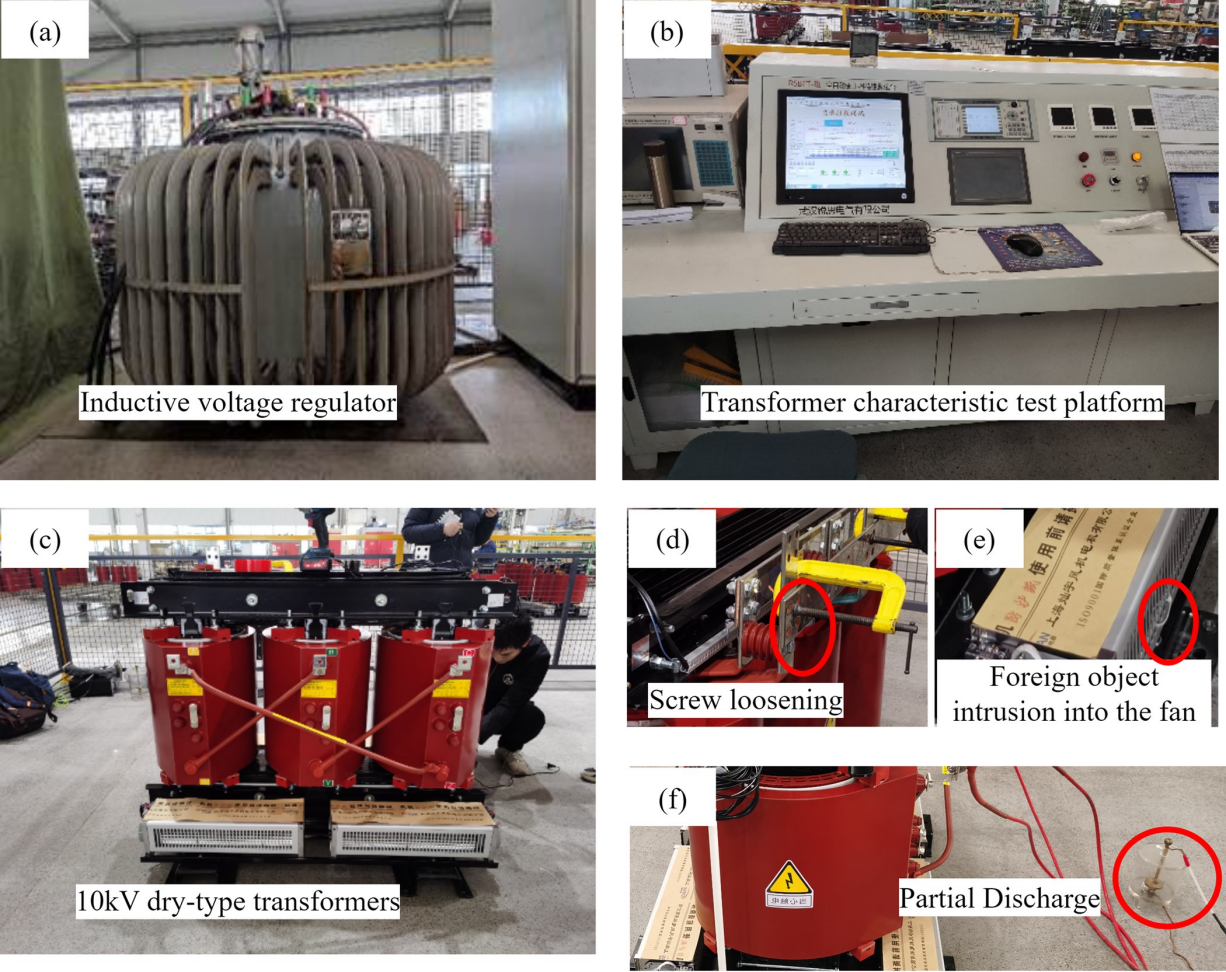
The main components of the defect simulation platform. (a) Inductive voltage regulator, (b) Transformer characteristic test platform, (c) 10kV dry-type transformer, (d) Foreign object intrusion into the fan, (e) Screw loosening, (f) Partial discharge.

The simulation test platform is controlled by a fully automatic transformer characteristic test bench. An induction regulator supplies power to the dry-type transformer. The induction regulator profile and parameters are presented in Fig 2(A) and Table 2. The transformer characteristic testbed is depicted in Fig 2(B). The dry-type transformer’s model number is SCB11-800, and its shape and parameters are shown in Fig 2(C) and Table 3. Three types of defects, foreign object intrusion into the fan, screw loosening, and partial discharge, are shown in Fig 2(D)-2(F). Since the induction regulator generates noise during operation, it must be placed at a distance from the dry-type transformer to prevent interference with the transformer’s sound signals.

**Table 2:** Parameter table of induction regulator.

Parameter	Value
Induction regulator model	T SJA-20/0
Output capacity (kVA)	200
Input voltage (V)	380
Load Voltage (V)	0–650
Maximum load current (A)	178
Operating frequency (Hz)	50
Cooling type	oil-immersed

**Table 3:** Parameter table of dry-type transformer.

Parameter	Value
Dry-type transformer model	SCB11-800
Size (mm)	1350×1000×1100
High voltage (kV)	10
Tap range	±2×2.5%
Low voltage (kV)	0.4
Coupling group	Dyn11
Standard short-circuit impedance	6%
No-load loss (W)	1360
Load loss (W)	6960

### 2.2 Voiceprint characteristics of different defects

Sound signals from various defect models are captured by a single MEMS microphone, preparing for the design of a microphone array. The microphone model used is IM69D120 [20]. According to temperature reliability tests conducted in accordance with standards such as JESD22 A-103E [21] and JESD22 A-108D [22], this microphone can operate and store data in environments ranging from -40°C to +125°C, demonstrating excellent temperature stability. This meets the temperature requirements of the IEC 61094–4 standard for microphones from -10°C to 50°C [23]. Therefore, the microphone can be used for dry transformer defect detection. The distance between the microphone and the dry-type transformer is set at 1 meter. The sampling rate is 48 kHz, and the duration of signal collection is 10 seconds. The sound pressure level is recorded by the Aiwa AWA-5636-0 sound level meter. Identifying characteristic patterns from time-domain signals is challenging, necessitating the use of Fast Fourier Transform (FFT) to convert signals to the frequency domain. The length of each FFT frame is set at 4096.

#### 2.2.1 Rated load test

**Fig 3:**
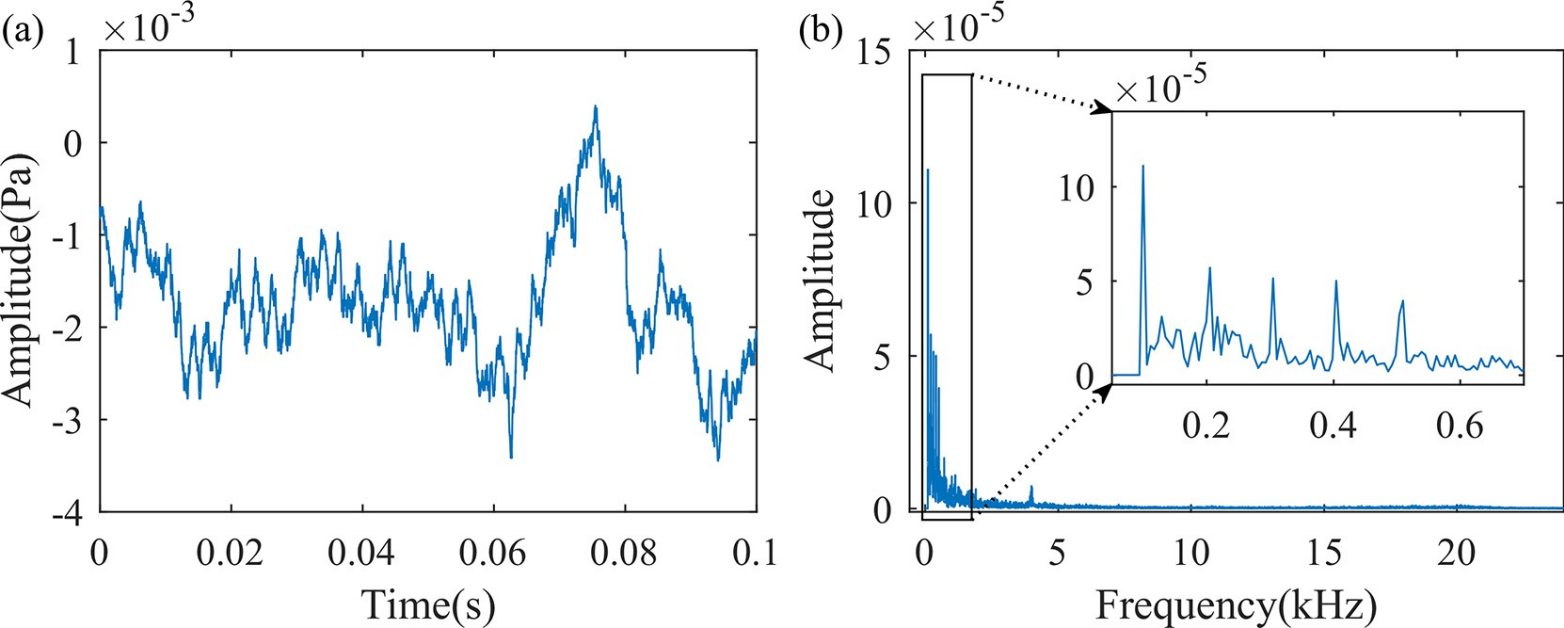
Sound signal of a dry-type transformer operating under rated load conditions. (a) Time domain waveform of 0.1s sound signal, (b) Frequency spectrum diagram of one frame of the sound signal.

When a short circuit occurs on the low-voltage side of the dry-type transformer, the current on the high-voltage side is gradually increased to its rated value. No significant sound is produced by the dry-type transformer. The sound pressure level recorded on-site is 49.0 dB. The time-domain sound signal is shown in Fig 3(A). The figure shows that the fluctuations in the signal do not follow a clear pattern. The spectrum of the signal, obtained by converting the time-domain signal to the frequency domain using FFT, is shown in Fig 3(B). This chart indicates that the sound signal of the dry-type transformer under rated load mainly has characteristic frequencies at 100, 200, 300, 400, and 500 Hz. This is due to the periodic vibration caused by the magnetostriction of the core during operation, which has a frequency of 100 Hz. Due to the unequal magnetic paths formed by the inner and outer frames of the core, frequencies that are multiples of 100 Hz, such as 200, 300, etc., also appear [24]. In addition, there is a smaller characteristic peak around 4000 Hz, with a very small amplitude, which may be related to the structure of the dry-type transformer itself.

#### 2.2.2 Foreign object intrusion into the fan

**Fig 4:**
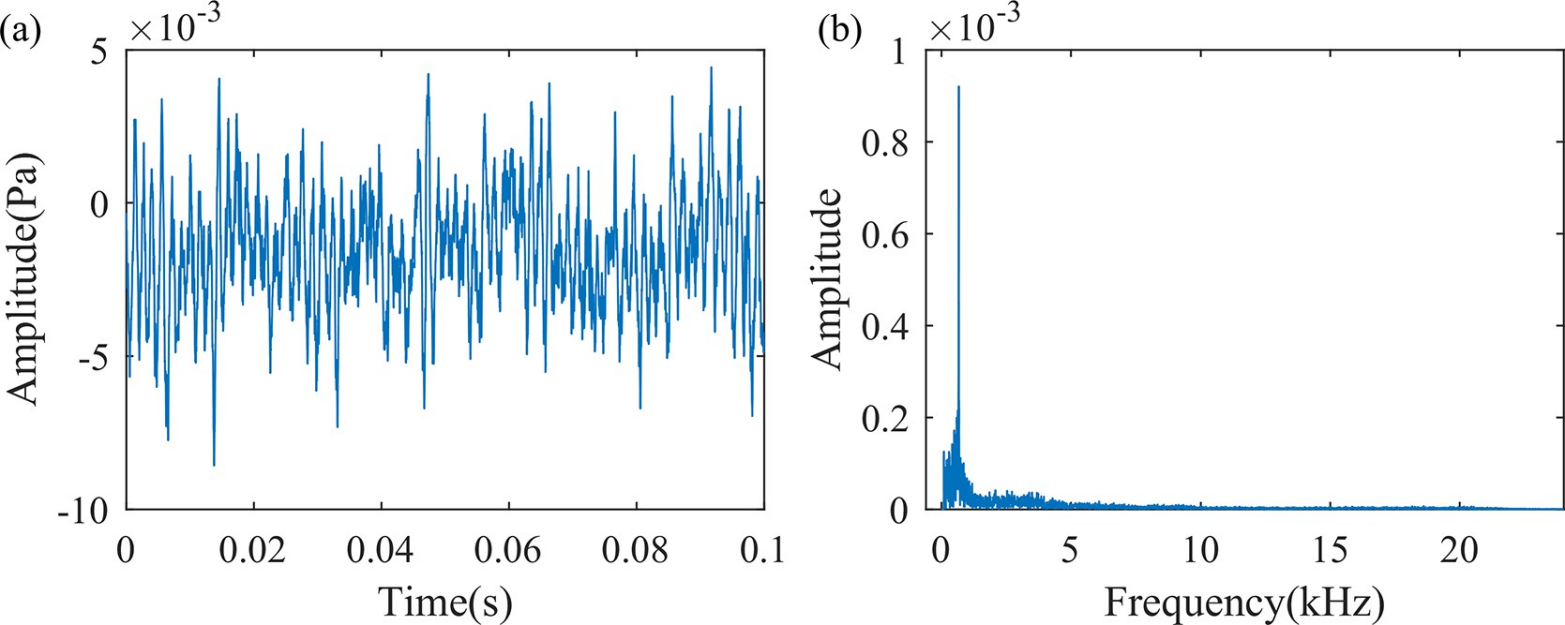
Sound signals during normal operation of the fan. (a) Time domain waveform of 0.1s sound signal, (b) Frequency spectrum diagram of one frame of the sound signal.

The sound signal collected when the cooling fan is operating normally is shown in Fig 4. There is a clear characteristic peak at 674 Hz, and the sound amplitude significantly increases. When the fan is operating normally, the sound at the air outlet is uniform and regular, with a sound pressure level of 61.0 dB.

**Fig 5:**
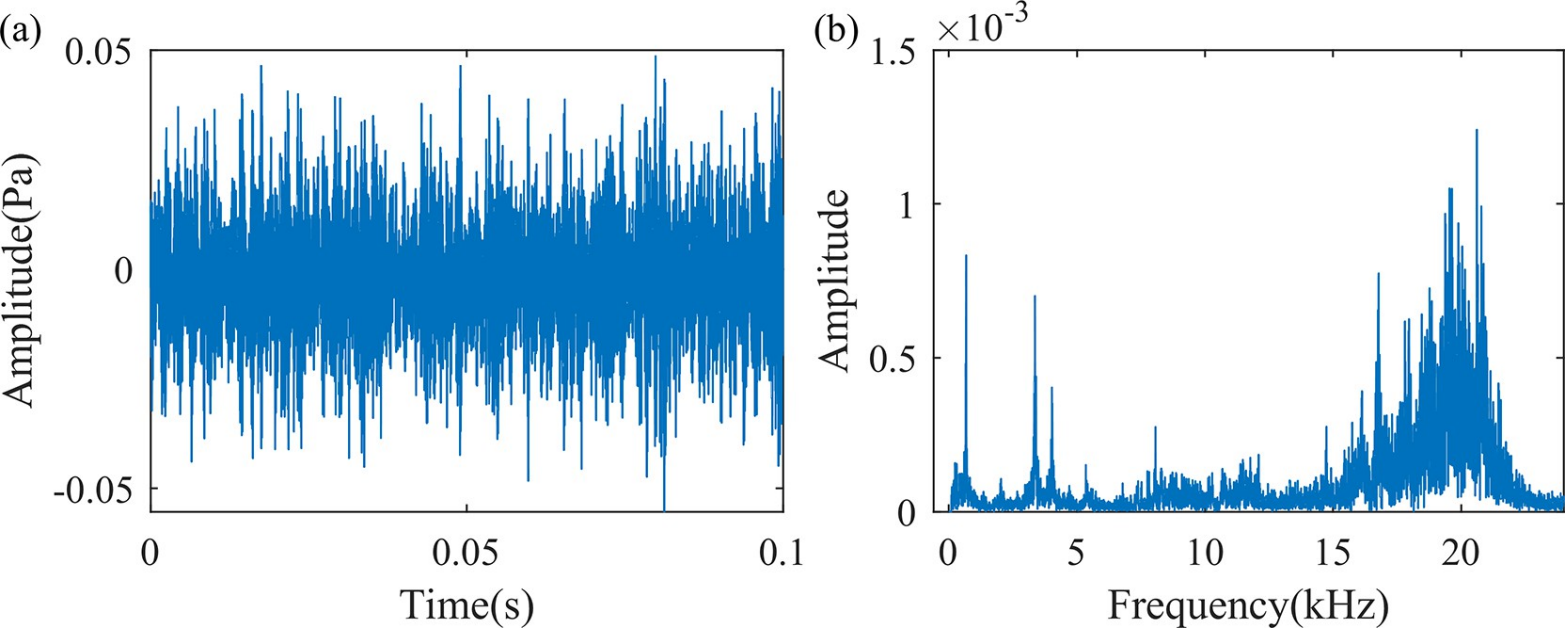
Sound signals when foreign objects invade the fan. (a) Time domain waveform of 0.1s sound signal, (b) Frequency spectrum diagram of one frame of the sound signal.

A sharp sound, with a sound pressure level of 68.2 dB, is caused by a defect of foreign object intrusion due to a plastic binding strap at the fan outlet. The signal waveform is shown in Fig 5. As can be seen from the figure, in addition to the characteristic peaks of the sound signal under normal working conditions, several characteristic peaks are enhanced. The first characteristic peak is at 679 Hz, which is almost the same as the characteristic frequency of the sound signal when operating normally. The amplitude of the characteristic peak near 20000 Hz exceeds the amplitude of the characteristic peak under normal operating conditions. This corresponds to the results measured by the sound level meter.

#### 2.2.3 Screw loosening

**Fig 6:**
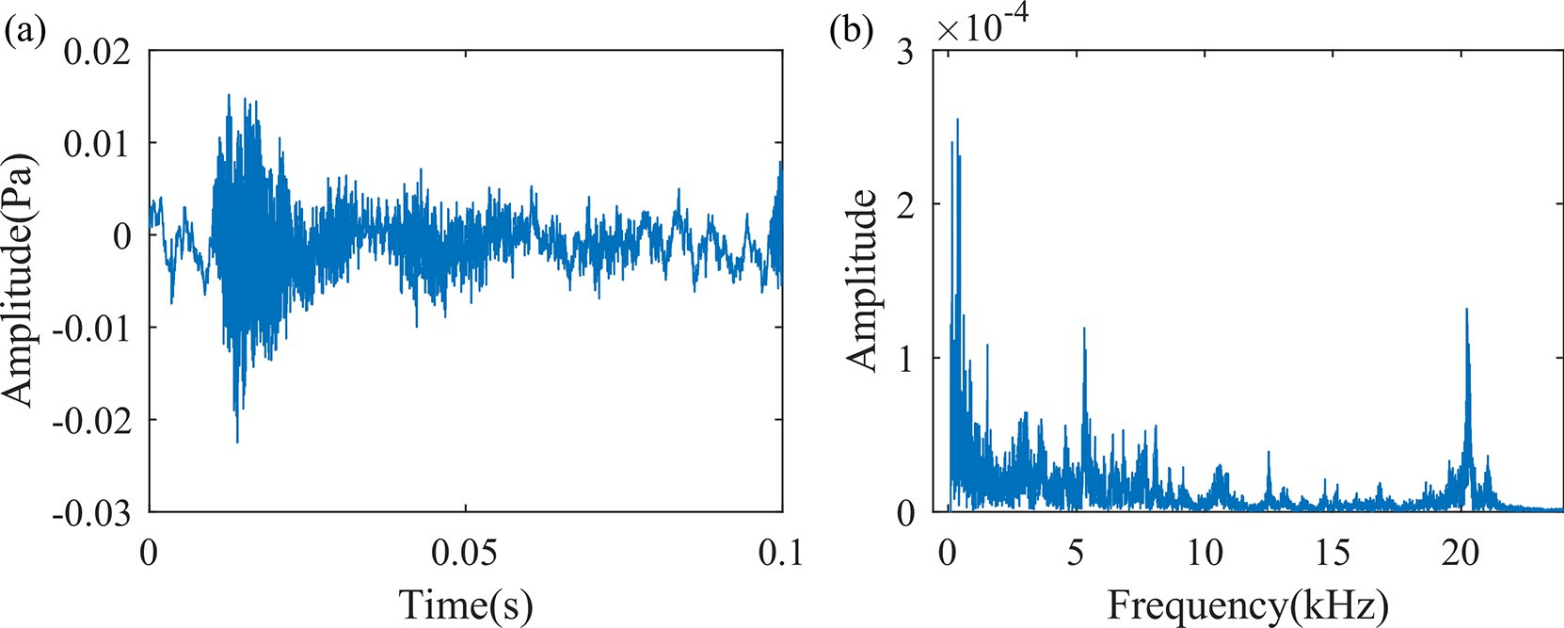
Sound signal when a screw loosening defect occurs. (a) Time domain waveform of 0.1s sound signal, (b) Frequency spectrum diagram of one frame of the sound signal.

Based on the rated load test, a screw loosening fault is located near the C-phase connector, as shown in Fig 2(E). A clear metallic collision sound can be heard, with a sound pressure level of 61.9 dB. The sound signal is shown in Fig 6. As can be seen from the figure, the sound signal produced by the screw loosening defect also has a rich characteristic frequency, with three clear characteristic peaks at 369 Hz, 5308 Hz, and 20230 Hz.

#### 2.2.4 Partial discharge

**Fig 7:**
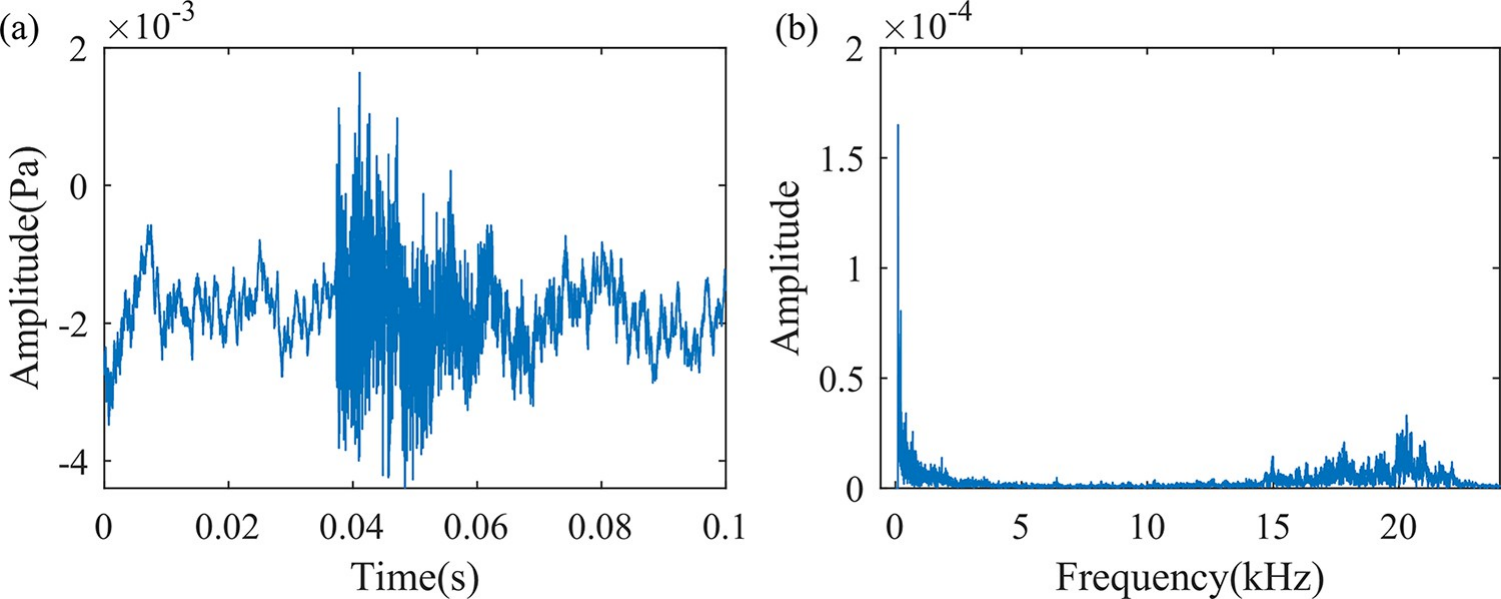
Sound signal when partial discharge defects occur. (a) Time domain waveform of 0.1s sound signal, (b) Frequency spectrum diagram of one frame of the sound signal.

A partial discharge defect between the A-phase and B-phase of the transformer shell is simulated using a needle-plate electrode, as shown in Fig 2(F). A faint air breakdown sound can be heard, with a sound pressure level of 47.2 dB. The sound signal of the partial discharge is shown in Fig 7. As can be seen from the figure, the characteristic frequency of the sound signal produced by the partial discharge shows a polarized trend, that is, the characteristic frequency is distributed at both ends of the measurable frequency band. There is a clear characteristic frequency band at 996–1839 Hz, and another clear characteristic frequency band in the range of 15000–22000 Hz.

## 3. Microphone array optimization design

The microphone array is an extremely important component of the acoustic imaging system, and its performance is affected by many factors, such as the number of microphones, array topology, application scenarios, sound source characteristics, manufacturing costs, etc. In this section, the PSO algorithm is used to design a 64-element, 8-arm planar spiral array by considering all of the above factors.

### 3.1 Acoustic wave propagation model

The acoustic propagation model can be divided into two models, near-field and far-field, and the critical values are generally calculated using Eq 1 [25].

$$L\leq \frac{2D^{2}}{\lambda }=\frac{2D^{2}f}{v}$$ (1)

where *L* is the distance from the array to the sound source, *D* is the array aperture, *λ* is the sound signal wavelength, *f* is the sound signal frequency, and *v* is the sound velocity in air.

When defects occur in a dry-type transformer, the characteristic frequency of sound is mainly distributed within the range of 680–20300 Hz. The critical distance, which falls between 1 and 29.9 m, is calculated using Eq 1. To ensure the microphone array can collect sufficient and distinct sound signals, the working distance between the microphone array and the dry-type transformer is set to 1 m. Based on this analysis, the acoustic wave propagation model is considered to be a near-field model.

### 3.2 Microphone array spatial structure

**Fig 8:**
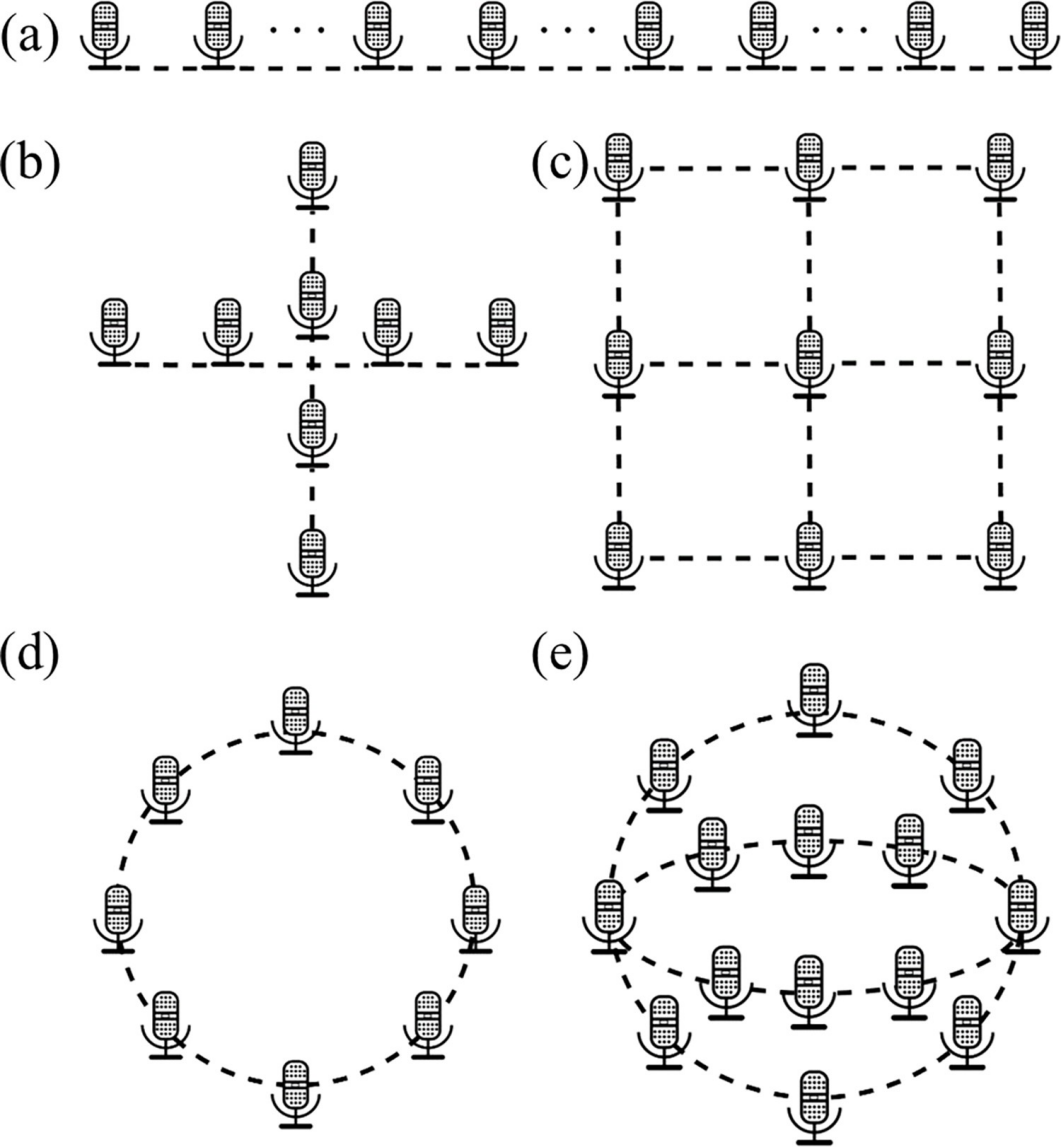
Typical spatial structures of microphone array.

Microphone arrays can be classified into 1D linear arrays, 2D planar arrays, and 3D stereo arrays according to their spatial structure [25, 26]. Some typical spatial structures of microphone arrays [25–28] are depicted in Fig 8. The 1D linear array can only be used to localize the directional angle of the sound source, and thus is not considered in this study. Both 2D planar arrays and 3D stereo arrays can achieve true acoustic imaging. However, 2D planar arrays have lower structural complexity, localization algorithm processing time, and other factors compared to 3D stereo arrays. They are easier to implement, more cost-effective, and more widely used [25–27, 29, 30]. Therefore, this paper designs a 2D planar array.

### 3.3 Number of microphones

Generally, the primary and secondary lobes in the acoustic cloud map can be used to evaluate the performance of the array [31]. The largest peak in the acoustic cloud map corresponds to the location of the sound source, which is typically referred to as the main lobe, while the remaining peak points are collectively referred to as side lobes. The narrower the main lobe, the higher the resolution, indicating that the array locates the sound source more accurately. The fewer side lobes and the lower their peaks, the stronger the array’s ability to suppress ambient noise.

From the characteristics of the sound signals of the dry-type transformer defects in Section 2, the characteristic frequency bands of the defects all include 4000 Hz. Therefore, 4000 Hz was chosen as the frequency of the sound source in the simulation design.

The relevant parameters in the simulation process are as follows: The geometric center of the rectangular array is the origin of the right-angle coordinate system (0, 0, 0), and the plane where the array is located is the xoy plane. The spatial coordinates of the single point source are (0.25 m, 0.25 m, 1.0 m). The localization algorithm is the classical beamforming algorithm. The scanning plane size is 1.2 m × 1.2 m, with a scanning interval of 0.02 m.

**Fig 9:**
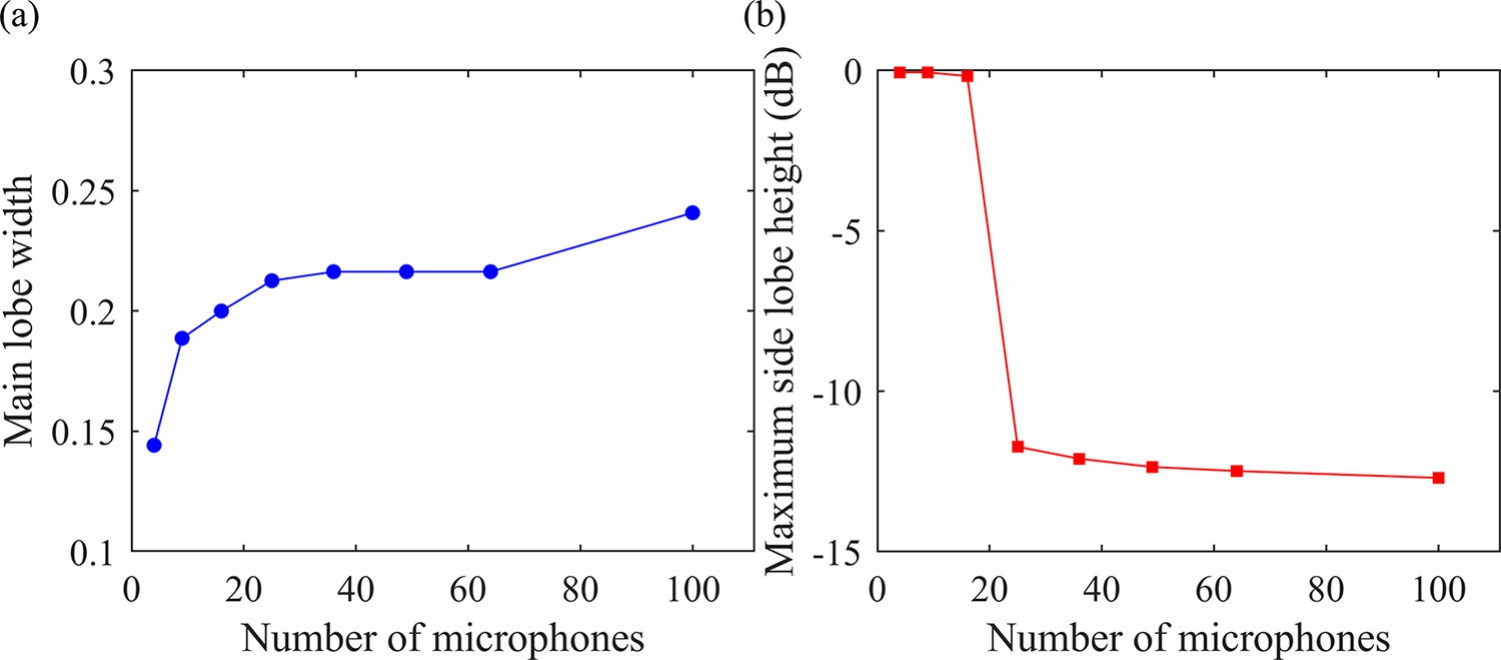
The effect of the number of microphones on the quality of acoustic imaging. (a) Main lobe width, (b) Maximum side lobe height (dB).

As shown in Fig 9, it can be observed that as the number of microphones increases, the main lobe width gradually increases and the side lobe levels gradually decrease. The main lobe width remains nearly constant when the number of microphones ranges between 36 and 64, while the side lobe levels gradually decrease. When the number of microphones exceeds 64, the main lobe width significantly increases, but the decrease in side lobe levels is less pronounced. Taking both the main lobe width and side lobe levels into consideration, a total of 64 microphones is selected.

### 3.4 Topology of the array

In addition to the 2D microphone array shown in Fig 8, the spiral array [30, 32] is also a common array structure.

A simulation study was conducted to investigate the impact of array topology on array performance. The spacing between array elements was set at 0.03 m, and the number of elements was 64. The spiral array used an Archimedean spiral to control element positions, as shown in Eq 2.

ρ=a+bθ (2)

where *ρ* is the radial distance between the helix and the coordinate center, *θ* is the rotation angle, parameter *a* controls the starting position of the helix, and parameter *b* determines the closeness between two adjacent helixes.

**Fig 10:**
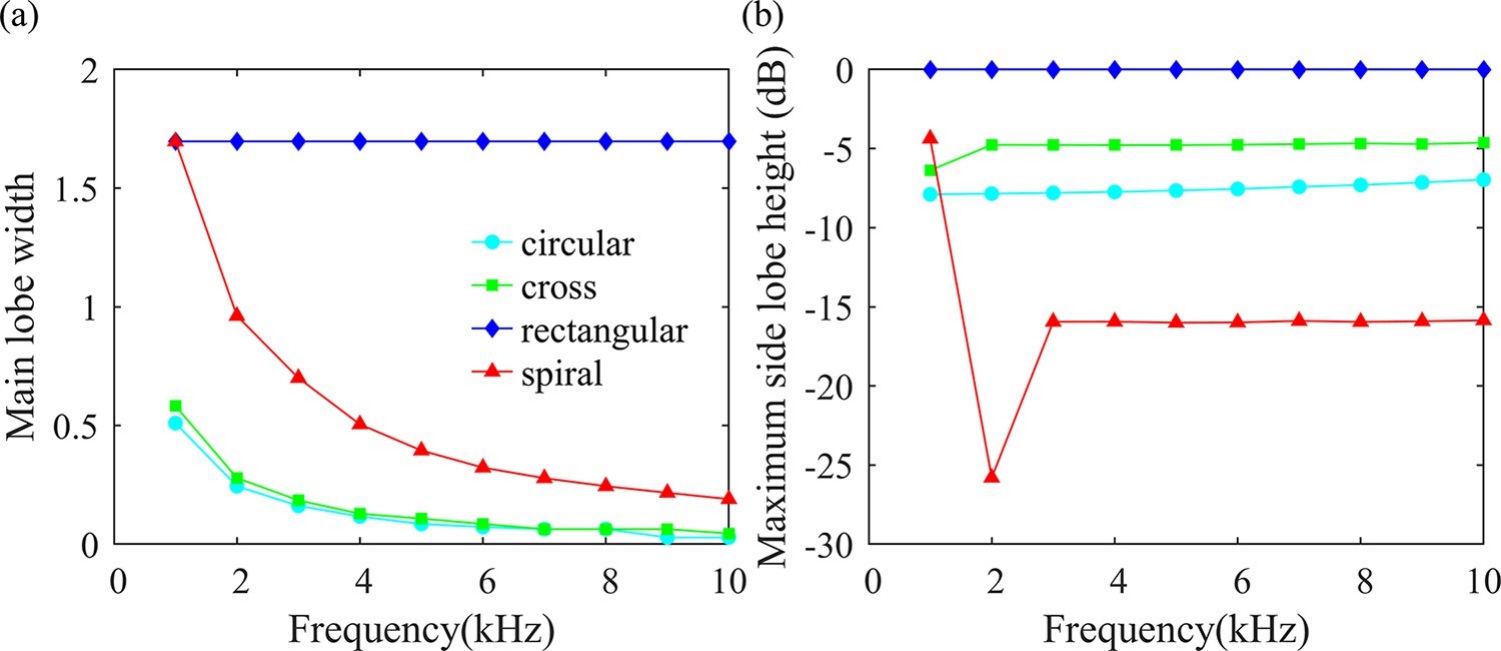
The effect of topology on the performance of microphone arrays. (a) Main lobe width, (b) Maximum side lobe height (dB).

The typical array performance simulation results are shown in Fig 10. As can be seen from the figure, the performance of the rectangular array is the worst. The beamwidth of circular, cross, and spiral arrays gradually decreases as the sound source frequency increases. The circular and cross arrays perform nearly identically well, followed by the spiral array. In terms of side lobe levels, the spiral array is the best, followed by the circular array, cross array, and rectangular array. The spiral array, due to its non-uniform distribution, can avoid repetitive spatial sampling and effectively mitigate spatial ambiguities, resulting in a significant reduction in side lobe height. Spatial ambiguities can easily lead to incorrect estimations of the number of sound sources. Therefore, overall, the spiral array’s performance is superior to that of other regular arrays. Thus, the spiral array is chosen as the topology for the array.

### 3.5 Microphone array optimization

**Fig 11:**
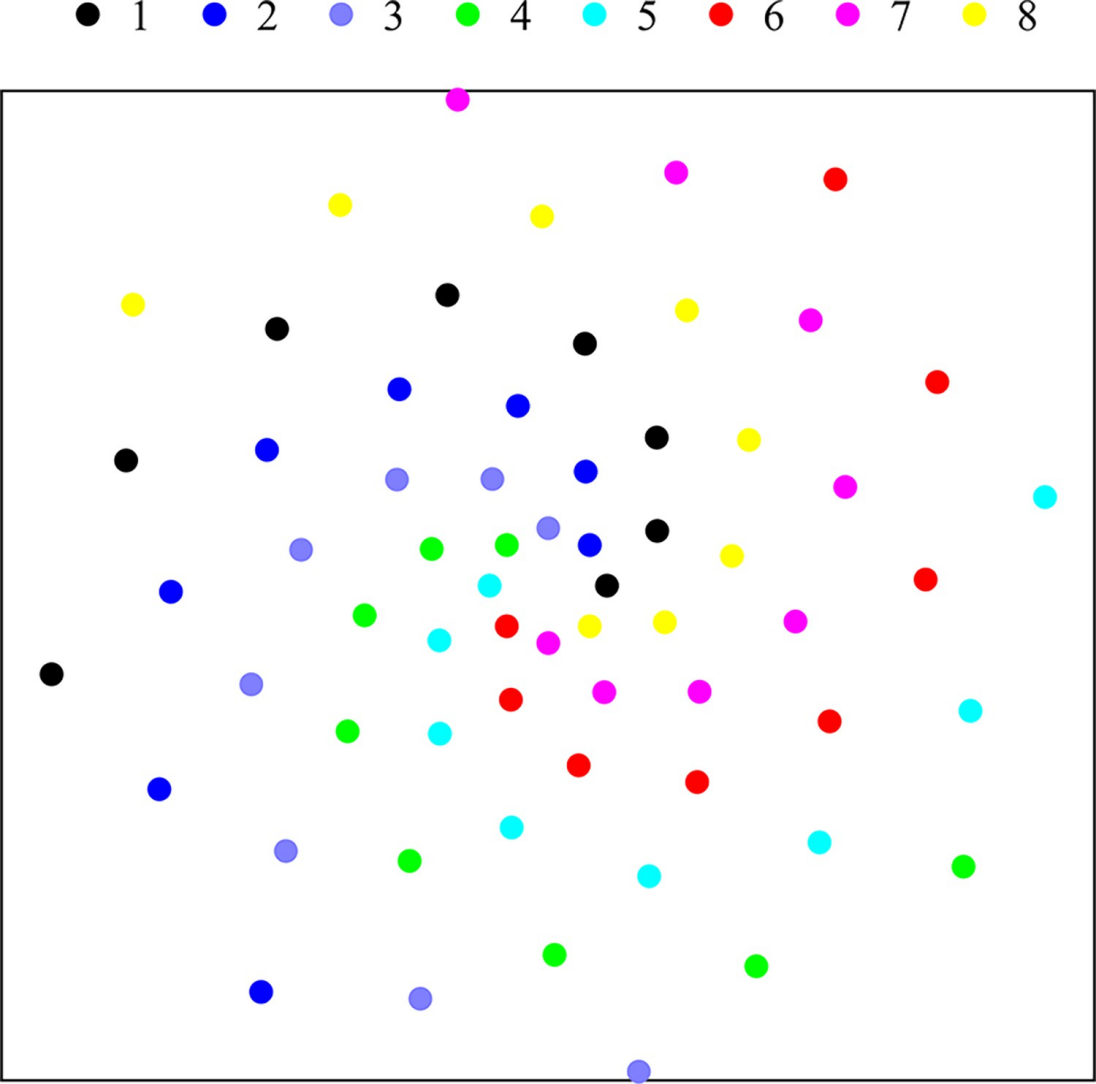
Schematic diagram of an 8-armed spiral array.

The performance of the helical array can be further enhanced by using a multi-armed helical structure [33, 34]. Based on our previous work [34], the 64-element multi-arm spiral microphone array has 8 arms. The first spiral arm is generated initially, and the other 7 spiral arms are created by rotating 45°, as illustrated in Fig 11.

In this paper, the PSO algorithm determines the positions of eight microphones on a single spiral arm. The position of the i-th array element is calculated as shown in Eq 3.


$$\left\{\begin{array}{rr} x_{i} & =(a+b(i-1)\frac{\theta }{8})cos((i-1)\frac{\theta }{8})\\ y_{i} & =(a+b(i-1)\frac{\theta }{8})sin((i-1)\frac{\theta }{8}) \end{array}\right)$$ (3)

Considering the array’s portability and practicality, the array aperture is set to D = 0.5 m. Consequently, the coordinate constraint of the ith array element on the kth spiral arm of the particle swarm algorithm is illustrated in Eq 4.


$$\{\begin{array}{cc} max(\left|x_{ik}\right|)-D/2 & <0\\ max(\left|y_{ik}\right|)-D/2 & <0 \end{array}$$ (4)

The fitness of the PSO algorithm is determined by the width of the main lobe and the maximum height of the side lobes. In this paper, the adaptation function is calculated as shown in Eq 5 [34].

$$F=k_{1}V_{\mathrm{main}}+k_{2}V_{\mathrm{beside}}$$ (5)

where *V*_main_ and *V*_beside_ denote the width of the main lobe and the maximum height of the side lobe, respectively, and *k*_1_ and *k*_2_ represent the scale factors, both set to 0.5 in this paper.

For the PSO algorithm, the number of particles is set to 30, the learning factors *c*_1_, *c*_2_ are set to 1.5, the inertia weight *w* is set to 0.8, the random numbers *r*_1_, *r*_2_ are within (0,1), and the maximum number of iterations is 150.

**Fig 12:**
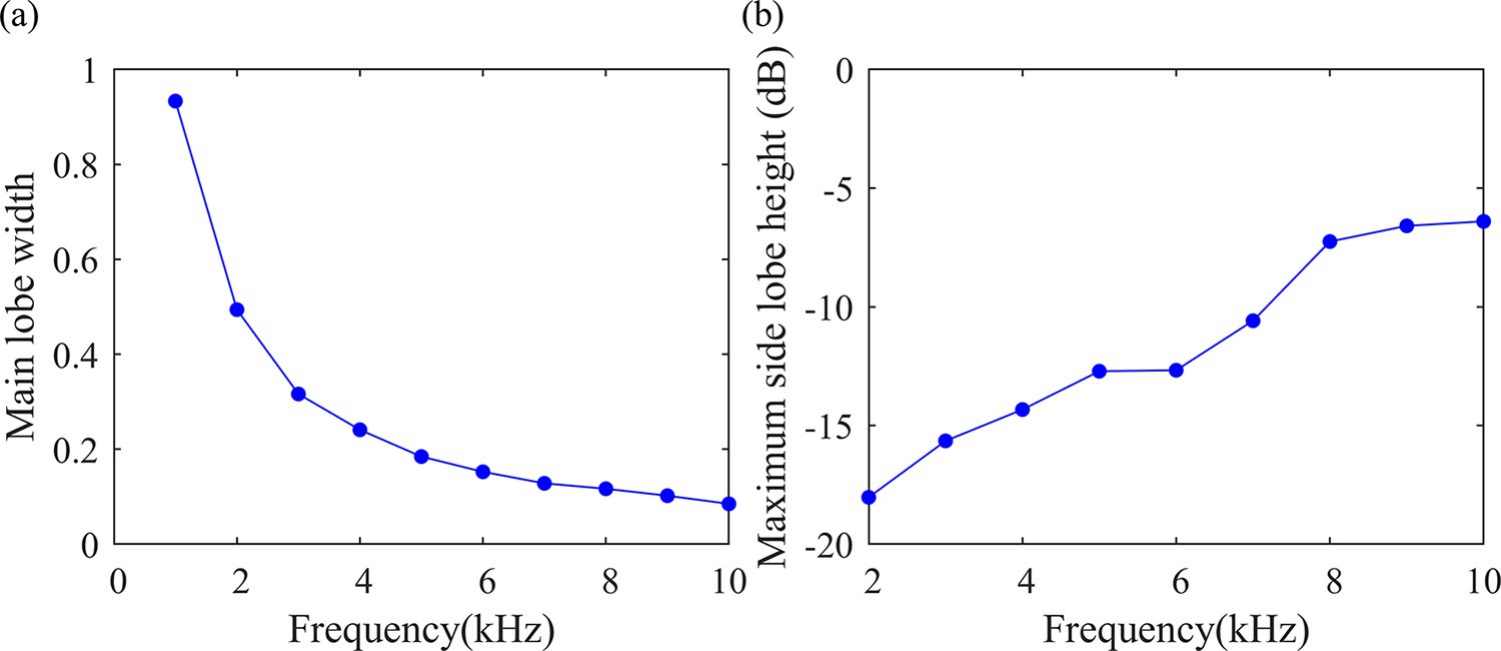
The performance curve of the optimally designed microphone array. (a) Variation law of the main lobe width with the source frequency, (b) Variation law of the maximum sidelobe heigh with the source frequency.

The cloud plots of the optimized array at different source frequencies are calculated, and the width of the main lobe and the maximum height of the sidelobes are shown in Fig 12(A) and 12(B). Compared to other arrays, the performance of the optimized array is superior at various frequencies. The most significant advantage is that the maximum height of the side lobes is below -5 dB in the range of source frequencies below 6 kHz, outperforming the other arrays in the previous section. According to Section 2, the effective characteristic frequency bands of the sound generated by typical defects of dry-type transformers include frequencies below 6 kHz. Therefore, the optimized multi-armed spiral array can achieve better acoustic imaging results.

### 3.6 Microphone array unit

**Fig 13:**
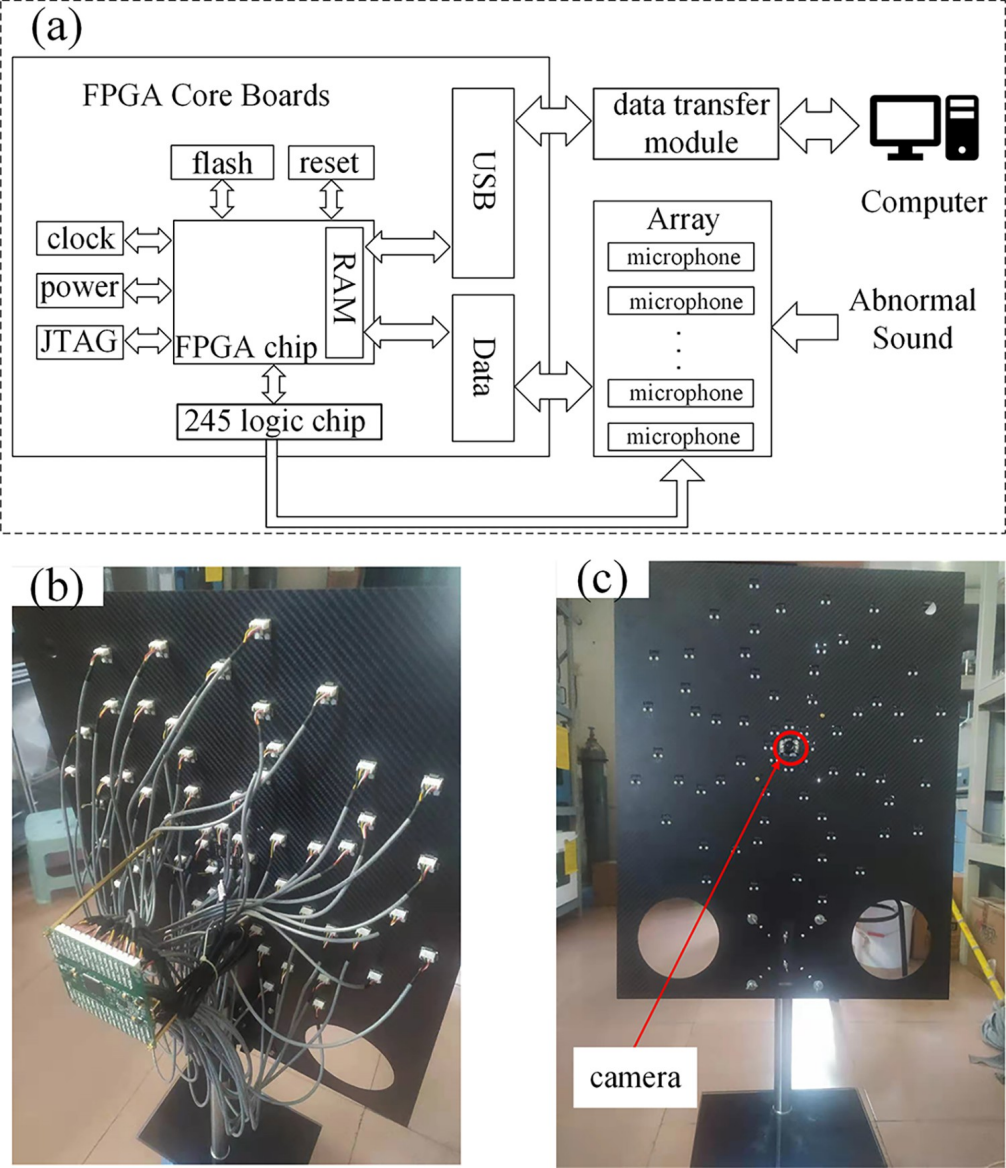
Schematic and physical drawings of the microphone array. (a) Schematic diagram, (b) Back of the hardware unit, (c) Front of the hardware unit.

The MEMS microphone model selected for this paper is IM69D120. The hardware system of an acoustic imaging device comprises a microphone array, an FPGA core board, and a data transfer module. In this study, the Cyclone IV series FPGA chip controls the MEMS microphone to collect sound signals through the 245 logic chip. The collected sound signal data is stored in RAM and subsequently transferred to a computer for processing via USB. The schematic and physical diagrams are depicted in Fig 13. A camera is installed at the center of the microphone array to enhance the visualization of the detection results, as shown in Fig 13(C). Optical photographs of the inspected object are taken during sound source localization. These optical photographs are fused with the acoustic cloud map to obtain a more visually informative acoustic imaging result.

## 4. Defect localization of dry-type transformers

### 4.1 Acoustic imaging algorithm

The mathematical expression of the sound signal collected by the microphone in the time domain is presented in Eq 6 [31].

$$s(t)=as_{0}(t)+n(t)$$ (6)

where **s**(*t*) represents the signal received by the array, *s*_0_(*t*) denotes the source signal, and **n**(*t*) refers to the sum of all ambient noise and the microphone’s background noise. **a** symbolizes the guiding vector of the array, as illustrated in Eq 7.


$$a={\left[\frac{1}{d_{1}}e^{-j(2\pi f\frac{d_{1}}{v})},\frac{1}{d_{2}}e^{-j(2\pi f\frac{d_{2}}{v})},\cdots ,\frac{1}{d_{M}}e^{-j(2\pi f\frac{d_{M}}{v})}\right]}^{T}$$ (7)

The sound signal collected in the field contains various information, such as the target source, environmental noise, and the microphone’s background noise. This reduces the localization accuracy of existing algorithms. According to the analysis in Section 2, sound signals with different defects have specific effective characteristic frequency bands. Hence, it is possible to intercept the required target frequency band for different defects, and the rest of the frequency band is not involved in the imaging calculation, thus suppressing interference. The multiple signal classification algorithm (MUSIC) offers higher spatial resolution [35]. Therefore, this paper improves the MUSIC algorithm.

The sound signal is converted from the time domain to the frequency domain using the Fourier transform, Eq 7 to Eq 8. At this stage, the frequency domain sound signal contains all frequency bands, from which the target frequency band containing the sound signal must be extracted. The extracted frequency domain signal is labeled as **s**_*g*_(*ω*). Its covariance matrix is calculated by Eq 9. An eigenvalue decomposition of the covariance matrix is performed to obtain the noise subspace. The covariance matrix can be decomposed into two parts related to signal and noise, as shown in Eq 10.

$$s(\omega )=as_{0}(\omega )+n(\omega )$$ (8)

$$R_{g}=E[s_{g}(\omega )s_{g}{\left(\omega \right)}^{H}]$$ (9)

$$R_{g}=U_{S}\Sigma_{S}U_{S}^{H}+U_{N}\Sigma_{N}U_{N}^{H}$$ (10)

where **U**_*s*_ is the subspace tensed by the eigenvectors corresponding to the large eigenvalues, i.e., the signal subspace, and **U**_*N*_ is the subspace tensed by the eigenvectors corresponding to the small eigenvalues, i.e., the noise subspace.

The equation for the spectral estimation of the MUSIC algorithm is given as Eq 11 [36].

$$P_{MUSIC}=\frac{1}{a^{H}U_{N}U_{N}^{H}a}$$ (11)

where **a** is the oriented vector of the array.

Considering that microphone damage may reduce localization accuracy, we employ a subarray superposition strategy to further optimize the imaging algorithm. The 64 microphones are divided into four non-overlapping groups, each containing 16 microphones. Three groups are used to form a sub-array. When performing acoustic imaging calculations, in addition to imaging the original 64-element main array, it is also necessary to image the 48-element sub-array. The main array and subarray power values are normalized and summed to form the final acoustic cloud map.

The overall computational flow of the improved MUSIC algorithm based on frequency domain features in this paper is shown in Table 4.

**Table 4:** Modified MUSIC algorithm.

Algorith-1
Input:
*M*∈***Microphone***: Coordinates of the microphone.
*S*∈***Signal***: Sound signals.
*X*_*i*_, *Y*_*j*_∈***Area***: The scanning range of the microphone array.
*X*_Step_, *Y*_Step_: Step of scanning.
Output:
*Y*∈P: Acoustic cloud map.
Start:
1: Initializing the coordinates of microphones.
2: Initializing the scanning range of the microphone array.
3: Receiving sound time domain data and converting it to frequency domain data using Fourier transform.
4: Extracting the target frequency band from the frequency domain data based on the characteristics of the abnormal sound.
5: Calculating the covariance matrix of the target frequency band according to Eq 9 and decomposing it into eigenvalues.
6: Deriving the noise subspace according to Eq 10.
7: Dividing 4 sub-arrays on the basis of the original microphone array.
8: Loop-1: for *m* = 1 to 5
9: Loop-2: for *i* = 1 to *X*_Step_
10: Loop-3: for *j* = 1 to *Y*_Step_
11: For the mth array, calculate the power value at the scan point (X_*i*_, Y_*j*_) according to Eq 11.
12: End Loop-3
13: End Loop-2
14: End Loop-1
15: Normalizing and summing the power values calculated for the five arrays and finally outputting the power values at different scanning grid points.
16: Connecting the power of each grid point and plotting the acoustic cloud.
End

### 4.2 Simulation verification

**Fig 14:**
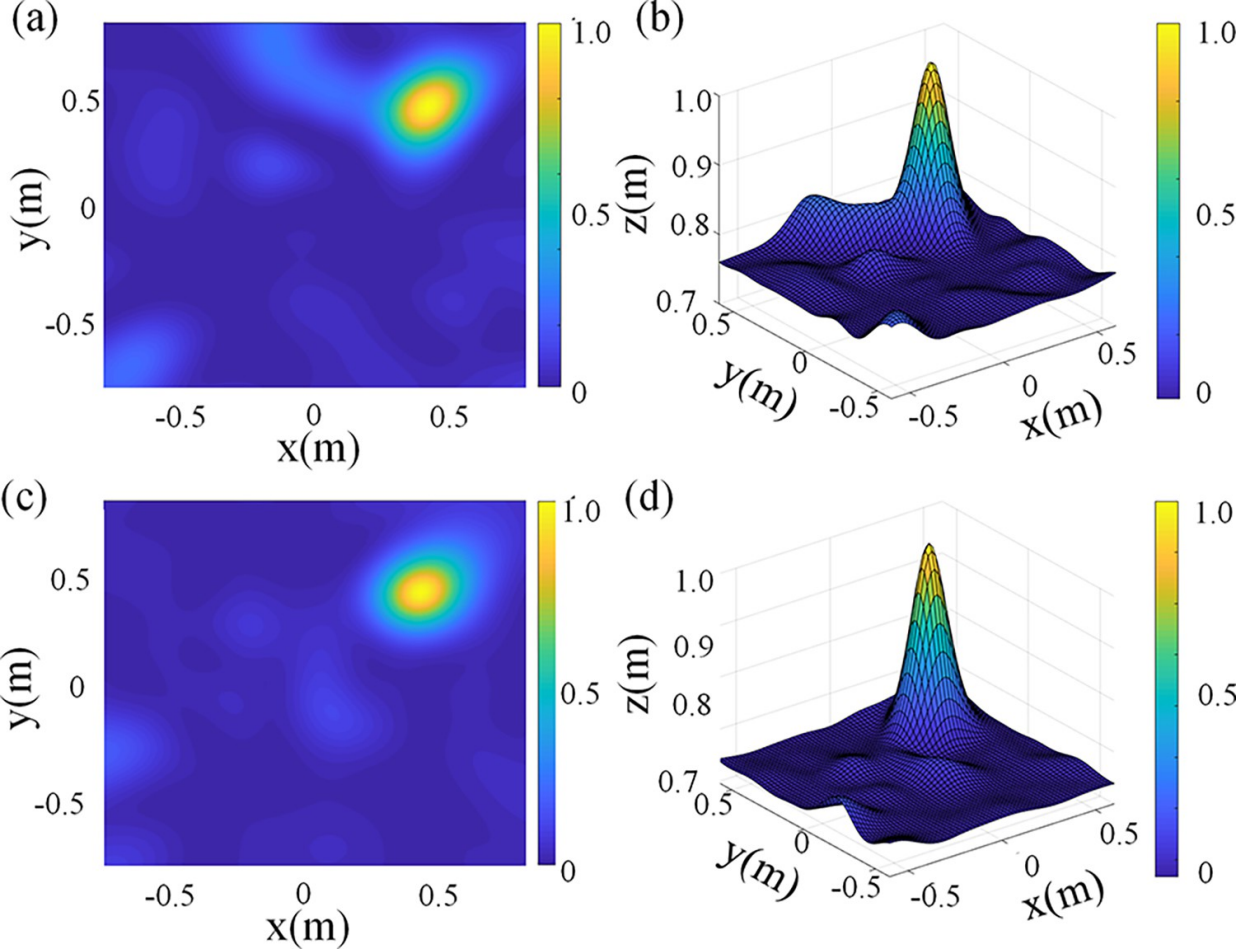
Cloud maps before and after MUSIC algorithm improved. (a) 2D cloud map generated by MUSIC algorithm (b) 3D cloud map generated by MUSIC algorithm (c) 2D cloud map generated by improved algorithm (d) 3D cloud map generated by improved algorithm.

The effectiveness of the improved algorithm is verified by simulation. The microphone array used is the optimized array from Section 3. The MUSIC algorithm and the improved MUSIC algorithm (referred to as the improved algorithm) proposed in this paper are used for acoustic imaging calculations, respectively. The results are displayed in Fig 14. It can be observed from the figure that after the improvement, the width of the main flap of the acoustic cloud map is reduced, and the maximum height of the side flap is decreased from 0.819 to 0.6499. The interference suppression capability of the improved algorithm is significantly enhanced.

### 4.3 Experimental verification

The defect experiments in Section 2 were repeated, and the defect localization was performed using the acoustic imaging device designed in Section 3.

#### 4.3.1 Foreign object intrusion into the fan

**Fig 15:**
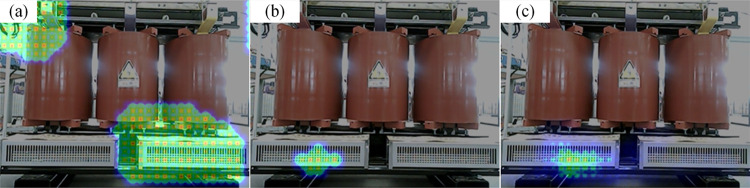
The localization results of the fan during normal operation. (a) Localization results of MUSIC algorithm, (b) Localization results of the improved algorithm, (c) Superimposed results of 20 frames of cloud map.

The acoustic images of the different algorithms during the normal operation of the fan are shown in Fig 15(A) and 15(B). The MUSIC algorithm cannot locate the sound source point, while the improved algorithm can locate the sound source at the location where the fan blade is rotating. The 20 frames of cloud maps from the improved algorithm are taken and superimposed, and the results are displayed in Fig 15. The localization area of the superimposed cloud map is larger than that of the single-frame imaging map, which indicates that the sound source will be offset at different moments. This is because the shape of the fan is long, and the maximum sound source point will appear at different locations of the fan outlet.

**Fig 16:**
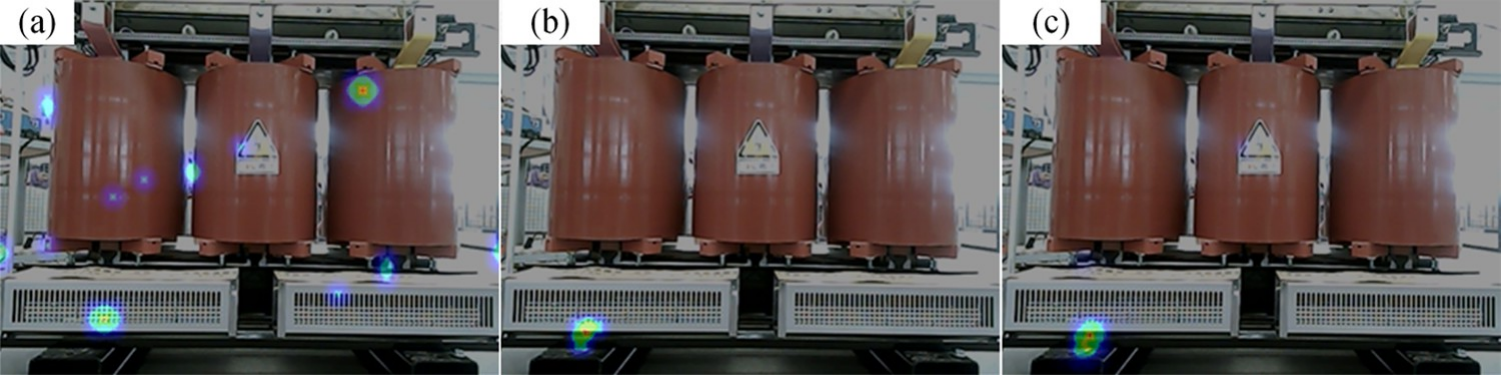
The localization results for foreign objects intrusion. (a) Localization results of MUSIC algorithm, (b) Localization results of improved algorithm, (c) Superimposed results of 20 frames of cloud map.

In the case of foreign body intrusion into the fan, the localization results are displayed in Fig 16(A) and 16(B). Multiple “fault points” appear in the MUSIC algorithm’s cloud map, making it difficult to locate the fault. The improved algorithm has only one “fault point” in the cloud map, which is consistent with the designed defect location. Therefore, the improved algorithm can accurately locate the location of foreign object intrusion into the fan. The 20 frames of the cloud map of the improved algorithm are taken and superimposed, as shown in Fig 16(C). The size and location of the “fault points” in the cloud are almost the same, which indicates that the improved algorithm has good stability. The localization accuracy after excluding the biased data is 0.013m.

#### 4.3.2 Screw loosening

**Fig 17:**
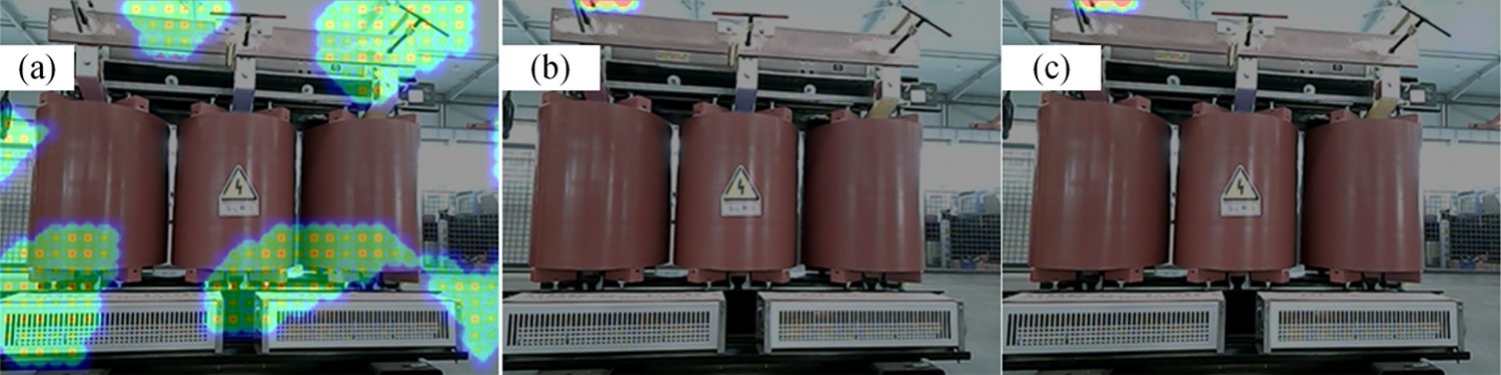
The localization results for screw loosening. (a) Localization results of MUSIC algorithm, (b) Localization results of improved algorithm, (c) Superimposed results of 20 frames of cloud map.

The acoustic imaging maps calculated by different algorithms are displayed in Fig 17. The improved algorithm can accurately locate the sound source, and the area of the calculated “fault point” is small, indicating its high localization resolution. This is because the sound intensity of the screw loosening defect is higher and corresponds to a higher characteristic frequency. The 20 frames of the cloud map of the improved algorithm are taken and superimposed, as shown in Fig 17(C). It can be seen from the figure that the size and position of the cloud map hardly changed, indicating that the optimization algorithm is very stable. The localization accuracy after excluding the biased data is 0.041m.

#### 4.3.3 Partial discharge

**Fig 18:**
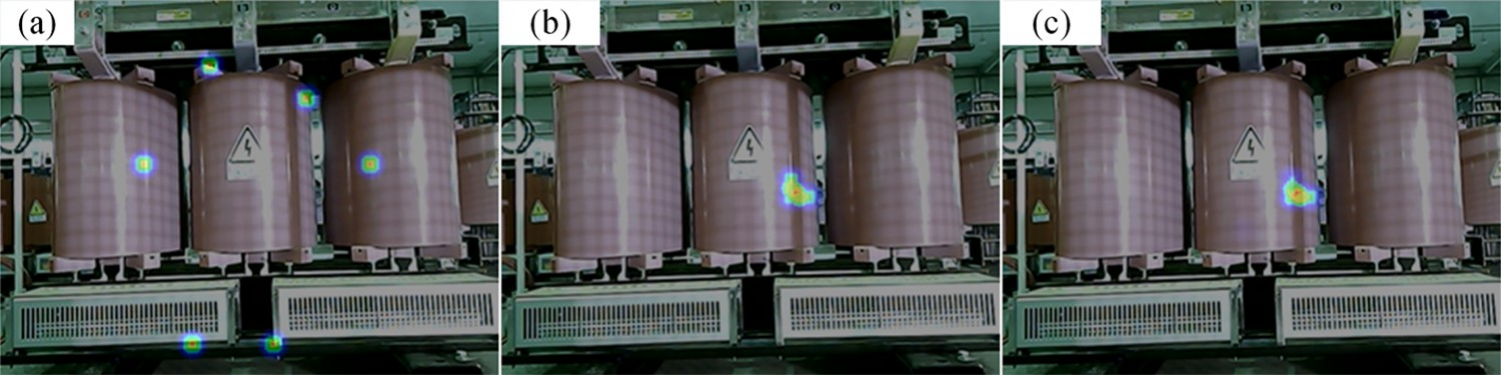
The localization results for partial discharge. (a) Localization results of MUSIC algorithm, (b) Localization results of improved algorithm, (c) Superimposed results of 20 frames of cloud map.

The acoustic imaging maps calculated by different algorithms are displayed in Fig 18. Several “pseudo-source points” appear on the cloud map of the MUSIC algorithm, making it difficult to locate the partial discharge. As a result, the improved algorithm can pinpoint the location of the partial discharge. The 20 frames of the cloud map of the improved algorithm are taken and superimposed, as shown in Fig 18(C). The superimposed cloud map is almost the same as the single-frame cloud map, which indicates the good stability of the improved algorithm. The localization accuracy after excluding the biased data is 0.013m.

## 5. Conclusions

### 5.1 Summary of findings

This study presents a detailed exploration of the localization method for typical defects in dry-type transformers using acoustic imaging technology. Distinctive techniques are employed, addressing challenges in sound and localization:

1. Acoustic characteristics of dry-type transformers: The findings indicate that the sound signal of these transformers under normal operation is subdued. The presence of defects leads to a discernible increase in sound intensity. In addition, each defect has a unique frequency band for sound source localization.
2. Efficient microphone layout strategy: The research reveals that enhancing the array’s performance is possible with the addition of microphones and an irregular spiral structure. A saturation phenomenon becomes apparent with the mere increase in microphone numbers. However, the spiral array, when optimized using the PSO algorithm, exhibits superior accuracy and exceptional interference rejection.
3. Enhancements to the MUSIC algorithm: Through the integration of time-frequency transformation and subarray power spectrum superposition, the localization ability of the MUSIC algorithm is markedly improved. This refined technique boasts notable stability and precision.

### 5.2 Limitation analysis

The abnormal noise defect diagnosis system developed in this study primarily validates its anti-interference capability in indoor settings. It has not undergone extensive testing in challenging outdoor environments, such as heavy rain or blizzards. One potential direction for future work is to further validate and enhance the equipment’s performance in more realistic settings.

Analysis from Section 2 reveals distinct voiceprint characteristics corresponding to different defects. These characteristics offer potential avenues for the classification and diagnosis of dry transformer defects. The classification method can refer to [14]. However, due to the scope and length constraints of this article, this area has not been explored in depth. Another potential direction for future research is to fully leverage the array of voiceprint data collected by the microphone array to improve the accuracy of dry transformer defect diagnosis.

In the realm of acoustic imaging technology, the localization capability is predominantly focused on sounds generated by defects within the 20Hz to 20kHz frequency range. Conversely, the UHF-based localization method is primarily designed to capture electromagnetic signals ranging from 300MHz to 3GHz. While commonalities in the localization algorithms are shared between both technologies, enhanced precision in localizing partial discharge defects is granted by the outstanding penetration and propagation attributes of UHF signals. Looking ahead to future developments, the integration of UHF technology with acoustic microphone technology into a hybrid array can be envisioned, aiming to achieve higher precision localization across a broader spectrum of fault types.
